# As cold as a fish? Relationships between the Dark Triad personality traits and affective experience during the day: A day reconstruction study

**DOI:** 10.1371/journal.pone.0229625

**Published:** 2020-02-25

**Authors:** Irena Pilch

**Affiliations:** Institute of Psychology, Faculty of Social Sciences, University of Silesia in Katowice, Katowice, Poland; Univeristy of Padova, ITALY

## Abstract

The Dark Triad of personality is a cluster of three socially aversive personality traits: Machiavellianism, narcissism and psychopathy. These traits are associated with a selfish, aggressive and exploitative interpersonal strategy. The objective of the current study was to establish relationships between the Dark Triad traits (and their dimensions) and momentary affect. Machiavellianism, grandiose narcissism, vulnerable narcissism and the dimensions of the Triarchic model of psychopathy (namely, boldness, meanness and disinhibition) were examined. We used the Day Reconstruction Method, which is based on reconstructing affective states experienced during the previous day. The final sample consisted of 270 university students providing affective ratings of 3047 diary episodes. Analyses using multilevel modelling showed that only boldness had a positive association with positive affective states and affect balance, and a negative association with negative affective states. Grandiose narcissism and its sub-dimensions had no relationship with momentary affect. The other dark traits were related to negative momentary affect and/or inversely related to positive momentary affect and affect balance. As a whole, our results empirically demonstrated distinctiveness of the Dark Triad traits in their relationship to everyday affective states. These findings are not congruent with the notion that people with the Dark Triad traits, who have a dispositional tendency to manipulate and exploit others, are generally cold and invulnerable to negative feelings. The associations between the Dark Triad and momentary affect were discussed in the contexts of evolutionary and positive psychology, in relation to the role and adaptive value of positive and negative emotions experienced by individuals higher in Machiavellianism, narcissism and psychopathy.

## Introduction

In recent decades, there has been an increasing number of studies showing that personality matters because it has important consequences for individuals and enables prediction of many life outcomes [1,2]. According to an evolutionary conceptualization, inherited personality traits are visible as behavioral tendencies and have environment-contingent fitness consequences [3]. Evolutionary researchers have found associations between fitness and general personality traits [4,5]. Various studies have demonstrated that even extreme values of personality traits can be adaptive in certain environmental contexts [3]. It has been shown that some personality syndromes that are often interpreted as disorders can improve fitness due to behavioral strategies that accompany them [6,7]. Such personalities are currently extensively investigated as the so-called the Dark Triad of personality [8].

The Dark Triad is a set of dispositions fostering the use of exploitative resource acquisition strategies [9], which enables the expropriation of other people’s resources using deception, manipulation, intimidation or coercion [10]. Research showed that the Dark Triad traits can increase fitness by reinforcing an exploitative, short-term mating (preferring many casual sex partners) [11,12] and, more generally, a fast life strategy (focused on getting immediate rewards and gratifications) [13]. However, persons with dark personalities can also incur some undesirable consequences of their strategy, such as mate defection [14], instability of interpersonal relationships [15], lower sexual satisfaction [16], different health problems [17], or receiving punishments for being identified as a cheater [18]. The potential individual costs of the Dark Triad behavioral strategy were also observable as lower subjective well-being declared by respondents [19,20]. In the current study, we aimed at exploring this issue by investigating daily emotional experience (which is also regarded as a key indicator of subjective well-being) in the context of individual differences in Machiavellianism, narcissism and psychopathy. The obtained relationships will be also interpreted in terms of evolutionary functions of emotions.

The adaptive functions of emotions rests on facilitating decisions of the allocation of behavioral effort through signaling the actual position of an individual (taking into account the state of environment and the condition of the organism) [21]. The role of emotions is to prepare an individual to respond optimally in situations that contain threats (negative emotions) or opportunities (positive emotions) [22]. Negative emotions seem particularly important because they “are defenses that help us to deal with situations that decrease fitness” [22] (p. 284). The particular emotional states may be treated as more specific programs that help individuals to cope with particular problems. In the current study, we concentrated on an analysis of the frequency and intensity of emotional states experienced in everyday life, categorized as positive or negative. It can be expected that some emotional tendencies (e.g., more frequent experiencing of negative affect, also observable as a trait negative affectivity or lower subjective well-being) [17,19,20], usually interpreted by psychologists as costs paid by individuals with the Dark Triad, can be also viewed as adaptive in light of an evolutionary theory.

The Dark Triad of personality–Machiavellianism, narcissism and psychopathy–is a cluster of three socially aversive personality traits [8]. The Dark Triad personalities share some common features, such as disagreeableness, low empathy, selfishness, duplicity, competitiveness and manipulativeness [9]. For many years, dark traits have been viewed as maladaptive by clinical and social psychologists. However, the terms “adaptive” and “maladaptive” have different meanings in psychology (promoting or diminishing health and well-being) than they do in evolutionary biology (enhancing or reducing fitness) [23]. Therefore, “biologically adaptive traits may or may not be socially desirable or conducive to health and well-being”[23] (p. 262). Recent research has often taken an evolutionary framework perspective when studying dark personalities [11,13–15,24]. This approach makes it possible to consider the Dark Triad behaviors in terms of potential advantages and discuss their adaptive values in different areas of functioning, including emotional experience in everyday life. Interest in this latter area has, to date, been limited, as highlighted in meta-analyses [25]. While some research has focused on dark personalities’ limited empathy [26–30] and emotional intelligence [31–35] or difficulties in emotion regulation [36–39], less attention has been paid to the broader examination of the Dark Triad and affective states in daily life. Addressing this gap, the present study explores associations between Machiavellianism, narcissism and psychopathy and momentary affective states.

The Dark Triad traits are often investigated as three unidimensional constructs. However, a growing number of studies analyze different types/dimensions of narcissism and/or dimensions of psychopathy [40–43]. The dimensional approach is particularly advantageous when affect is of interest because the variants of narcissism and psychopathy show opposing relationships with emotionality [44–49]. Such an approach was used in the present study.

Machiavellianism is a trait defined by manipulative and exploitative interpersonal style. According to Christie and Geis [50], “cool syndrome” (coldness and detached affect, being cool and rational in social situations) is a central feature of Machiavellianism. “High Machs” are described as cynical and misanthropic, with a general tendency to emotional coldness, which can help them to manipulate and exploit others. They “show less emotionality and have fewer affective reactions than other people do towards situations, others, the self, and moral issues” [51] (p. 396). However, positive correlations between Machiavellianism and neuroticism, emotional instability and susceptibility to stress [52] suggest that Machiavellian coldness may partly be “in the eye of the beholder.” Research shows that Machiavellianism is associated with alexithymia [53], difficulties in expressing emotional states [39] and is inversely related to emotional well-being [17].

Narcissism, when treated as a trait, is connected with self-love, self-absorption, a sense of superiority, and attention seeking. Research suggests that there are two variants or dimensions of narcissism: grandiose and vulnerable [44]. Grandiose narcissism is characterized by egocentrism, grandiosity, entitlement, aggression and dominance. Grandiose narcissism is also connected with extraversion, emotional resilience, self-confidence and higher declared well-being. Cross-sectional research has demonstrated a positive relationship between grandiose narcissism and positive affectivity [45,54] and a negative relationship with depression and neuroticism [55,56]. Vulnerable narcissism, on the other hand, is related to self-absorption, defensiveness, introversion, a tendency to hold unrealistic expectations, and having a fragile self-confidence. Vulnerable narcissism has been associated with neuroticism [57,45], negative affectivity [45,46], depressive and anxious temperament [58], and negatively associated with positive affectivity [45,46].

The most recent three-dimensional conceptualizations of narcissism claim that narcissism has a more complex structure [59–61]. These models supplement narcissistic grandiosity and vulnerability with the third dimension to capture their common components. In the Narcissism Spectrum Model [62], the following dimensions of narcissism are distinguished: entitled self-importance, which is the main characteristics of narcissism, and the two nearly orthogonal factors–narcissistic grandiosity and narcissistic vulnerability. In turn, the Trifurcated Model of Narcissism [63,64] proposes such dimensions as agentic extraversion, narcissistic neuroticism and self-centered antagonism (the “core” of narcissism). Despite the differences in the names of components of narcissism, the integrative models of narcissism seem congruent [64]. Both models have received empirical support [61,65,66].

In the present study, the Narcissistic Personality Inventory (NPI-13) [67,68,64] total score was used to assess grandiose narcissism and the Hypersensitive Narcissism Scale (HSNS) [57] was applied to measure vulnerable narcissism. Both questionnaires can be considered valid measures of narcissistic grandiosity and vulnerability, respectively [59,69]. Additionally, the scores on the sub-scales of the NPI allow distinguishing more antagonistic element of grandiosity (Exploitativeness/ Entitlement).

Finally, psychopathy is connected with many serious dysfunctions. The main features of psychopathy are high levels of callous and unemotional traits (e.g., lack of empathy, emotional detachment, shallow affect, incapacity for love). Research suggests that psychopathy may be a heterogeneous construct, with two or three variants [70]. Primary psychopaths have a limited ability to feel some emotions, such as fear, anxiety or guilt, that may be visible as lower negative affect; they also show higher levels of extraversion, which is in line with positive relation of primary psychopathy with positive affectivity [47–49,71]. However, other studies reported no relationship between primary psychopathy and positive affectivity and a positive association of primary psychopathy with negative affectivity [49].

The secondary variant of psychopathy is associated with impulsivity, depression, higher emotional distress, negative affect and lower positive affect [71,47,49]. The important difference between these two variants of psychopathy is in their affective deficits and anxiety/ neuroticism: primary psychopaths are deficient in emotionality and have low anxiety, while secondary psychopaths have fewer affective deficits and higher anxiety [49] (p. 529). These opposing relationships of primary and secondary psychopathy with emotionality can make correlations between emotionality and overall psychopathy non-significant [72].

The triarchic model of psychopathy [73–75] is the most current attempt to resolve the issue of multidimensionality. It includes three interrelated but distinct phenotypic constructs: meanness, boldness and disinhibition. Given the differences between them, it is useful to analyze them separately [75] (p. 360). Disinhibition is related to impulsiveness, impaired affect regulation, negative emotionality, hostility, mistrust and aggression. Meanness is defined by low empathy, callousness, excitement seeking, predatory exploitativeness, destructiveness and problems with maintaining close relationships. Boldness is connected with low anxiety, emotional resilience, interpersonal effectiveness, assertiveness and reflects more “positive” features of psychopathy. Thus, the triarchic model includes both adaptive and maladaptive aspects of psychopathy, which is especially important when sub-clinical groups are investigated. This model was used in the present study.

The aim of the current study was to establish relationships between the Dark Triad traits and momentary affective states in order to facilitate a clearer understanding of the specificity of daily affective experiences in people with dark personalities. Affect defined as “the conscious subjective aspect of emotions” [76] (p. 839) is typically measured by self-reports. Adopting a dimensional approach to affect [77–79], our study focused on the two basic affect dimensions: “positive affect” (i.e., experiencing pleasant emotions) and “negative affect” (i.e., experiencing unpleasant emotions), which can be assessed either as a state or as a trait [78]. These dimensions of emotional experience are congruent with those used in a number of evolutionary studies considering the adaptive functions of emotions [22]. The dimensions of affect can be measured using multi-item methods [80,81]. However, when subjects are asked to fill in questionnaires repeatedly (e.g., in diary or day reconstruction studies), short lists of emotional words or pictures [82,83,84] or one-item measures [85,86] can be more appropriate [87]. For this reason, we decided to use a short list of emotional words in our study.

In the current study, we followed the suggestion of Sleep, Lynam, Hyatt and Miller [88] (p. 947) that zero-order approaches should be prioritized when studying the Dark Triad constructs. Our main focus was on the bivariate relationships between affect and the Dark Triad traits: does momentary affect vary as a function of the particular Dark Triad traits? In order to examine this, we formulated the following hypotheses. First, both the results of cross-sectional studies and some features of the construct (e.g., negative world views and a negative cynical attitude toward people, which may be a source of distress) suggest that Machiavellianism would be positively associated with momentary negative affect (NA) and negatively associated with momentary positive affect (PA; Hypothesis 1). Second, we put forward a hypothesis on a positive association of grandiose narcissism with momentary PA and a negative association with momentary NA (Hypothesis 2). However, we postulate that vulnerable narcissism will be positively related to momentary NA and negatively related to momentary PA (Hypothesis 3). Finally, taking into account the fact that meanness and disinhibition are related to primary and secondary psychopathy [75], and boldness is related to grandiose narcissism [73,89], and considering the results of recent research on triarchic psychopathy dimensions [90–92], we hypothesize that: boldness will be positively related to momentary PA and negatively related to momentary NA (Hypothesis 4); disinhibition will be negatively related to momentary PA and positively related to momentary NA (Hypothesis 5), and meanness will be negatively related to momentary NA (Hypothesis 6).

## Materials and methods

### Participants and procedure

A group of 286 university students (109 males, age *M* = 21.3, *SD* = 1.8) was recruited through advertisements on campuses and on social media from a large university in Poland. Inclusion criteria included age (18 years and above) and consent to participate. Sixteen persons (5.6%) had incomplete questionnaires and were excluded from the analysis. Thus, the final sample consisted of 270 persons (104 males, 38.5%).

The study was voluntary and without compensation. Participants did not provide any personal data and a coding system for the questionnaires was used. Additionally, they returned the questionnaires in sealed envelopes. They were also assured that there are no wrong answers and that all their opinions are important. Participants were informed that the study was designed to explore the relationship between personality and daily emotions. No written consent was obtained because all the participants were volunteers. Oral consent to participate was obtained prior to participation. The current study received approval from the Ethics Committee of the Faculty of Pedagogy and Psychology, University of Silesia in Katowice.

For this study, the participants were split into groups of 10–15 persons, and sessions were run in university lecture rooms. They were provided with oral and written instructions. Each group was accompanied by two experimenters. The survey lasted 70–90 minutes and was divided into two parts with a break in between. In part 1, the participants provided socio-demographic data and filled out measures of the Dark Triad traits. In part 2, they completed a day reconstruction questionnaire.

#### The day reconstruction method

The Day Reconstruction Method (DRM), developed by Kahneman, Krueger, Schkade, Schwartz and Stone [93] as an alternative to the Experience Sampling Method (ESM) [94], is an experiential measure of affect. Participants are asked to divide their previous day into distinct episodes, list all the episodes, describe their features (e.g., what he or she was doing), report the time that each episode began and ended and evaluate affective states that they experienced during each episode. This technique can reduce memory and aggregation biases and it is easier and less time-consuming for a participant than the ESM (as only a single session is required). The stability and validity of measures of affect assessed by the DRM were confirmed in previous research [95–98].

To avoid respondents’ concentration on more salient or memorable events, the DRM proposes two separate phases of the survey. In the first phase, participants are asked to remember and describe in detail what they did yesterday. The aim of this phase is to reconstruct a previous day as thoroughly as possible. All events (“episodes”) should be described. Episodes are discrete activities, such as eating a breakfast, commuting to work or school, writing a report, or socializing with friends [93]. Participants are aware that they are preparing this “diary” for themselves i.e., they will not have to show it to anyone. After preparing a diary the second phase begins (i.e., participants answer the questions about affective states that have been experienced by them during each episode described earlier in diaries).

The questionnaire used in the current study was similar to that proposed by Kahneman et al. [93,99]. In section one, the participants were asked to construct a diary listing all activities/episodes they engaged in throughout the previous day, and to write down the beginning and the end of each episode. In section two, the subjects were requested to answer a series of questions for each episode, including (1) when the episode began and ended, (2) what they were doing (making a choice from 15 options), (3) who they were with (eight options), and how they felt in this situation (six affect dimensions). The subjects described, on average, 11.3 episodes (*SD* = 3.4, *Me* = 12, range 3–18), which gave 3047 measurements.

### Measures

#### Momentary affect

A list of adjectives was used to assess emotional states experienced during the day.

The adjectives were selected from the circumplex models of affect [100–103] that organize affective states in a two-dimensional circular structure. The two dimensions (pleasantness and arousal) form four quadrants of affect. As pleasantness (positivity) was of interest in the present study, three “positive” affect words (i.e., pleasant affective states: ‘happy’, ‘enthusiastic’, ‘relaxed’) and three negative affect words (i.e., unpleasant affective states: ‘annoyed’, ‘afraid’, ‘depressed’) were selected. Among these adjectives, two adjectives (‘relaxed’, ‘depressed’) are located in the low-activation quadrants of the model whereas the remaining adjectives are located in the high-activation quadrants. The negative affect words are related to three basic emotional states (anger, sadness and fear) that are recognized by the vast majority of theories of emotions. Short lists of adjectives describing emotions were previously used in many studies to measure affect [82,104–106].The participants described their affective states (e.g., “I felt happy in that situation”) using a 7–point scale (1 = “not at all”, 7 = “extremely”).

Dimensional approach to investigating emotions was used in the current study, thus, the two indexes (for positive and negative affect) were calculated. The principal-components factor analysis was conducted in order to check whether the relationships between positive affective states and negative affective states were strong enough and whether separate negative and positive affect indexes can be calculated. A similar approach was previously used in other studies that adapted the dimensional approach to emotions [107–109]. The analysis identified two factors with eigenvalues greater than 1 that explained 76% of the momentary affect variance. Factor loadings after Varimax rotation reveal that all the positive affect items loaded strongly on the first factor (>.76) and all the negative affect items loaded strongly on the second factor (>.62). The scores on the items ‘happy’, ‘enthusiastic’ and ‘relaxed’ were averaged to form a momentary positive affect (PA) index (*α* = .87), whereas the average of ratings on ‘annoyed’, ‘depressed’, and ‘afraid’ created a momentary negative affect (NA) index (*α* = .77). The affect balance (“net affect”) score was calculated by subtracting momentary NA form momentary PA for each assessment [110]. The score can vary from –6 (lowest affect balance) to 6 (highest affect balance).

#### The Dark Triad measures

A Polish version of the Mach IV [50,111] was used to measure Machiavellianism (20 items; 1 =“fully disagree,” 7 =“fully agree”; *α* = 0.74). A Polish validated version of the Narcissistic Personality Inventory (NPI-13) [67,112] was used to assess grandiose narcissism (13 items; 1 =“fully disagree,” 7 =“fully agree”; α = 0.64). The NPI-13 consists of three sub-scales: Leadership/Authority (LA; 4 items, *α* = 0.6), Grandiose Exhibitionism (GE; 5 items, *α =* 0.7), and Entitlement/Exploitativeness (EE; 4 items, *α =* 0.2). The results regarding Exploitation/Entitlement were not interpreted because of very low reliability of this sub-scale.

Vulnerable narcissism was assessed with a Polish version of the Hypersensitive Narcissism Scale [57,113] (HSNS; 10 items; 1 = “strongly disagree,” 5 = “strongly agree”; *α* = 0.57). Triarchic psychopathy was measured with the TriPM-41 [114] (41 items; 0 = “false,” 3 = “true”; boldness 15 items, *α* = 0.79; meanness 10 items, *α* = 0.83; disinhibition 16 items, *α* = 0.76), a shortened Polish validated version of the Triarchic Psychopathy Measure (TriPM) [74]. The TriPM-41 has good psychometric properties and was previously used in several studies [16,41].

### Statistical analyses

The data from the current study have a multilevel structure and were analyzed with a series of multilevel random coefficient models [115]. We examined relationships between momentary affect and the Dark Triad traits within an aggregationist model where “observations are nested within persons, and relationships between these means (intercepts from level 1) are examined at level 2” [116] (p. 805). According to Nezlek [116], such analyses are more accurate than ordinary least squares analyses because they use a procedure of ‘precision weighting’ (the intercepts are weighted at level 2 by the number of observations and the consistency of responses). All of the variables were standardized to enable a comparison of coefficients, which can be interpreted as standardized regression coefficients in ordinary least squares analyses [117] (p. 781). The HLM-7 program [118] and the restricted maximum likelihood method of estimation were used. Fixed effects with robust standard errors were estimated. All the coefficients were modelled as random. The models were adjusted for participant sex.

We first analyzed whether participants differed in their average levels of reported momentary affect. Afterwards, a series of analyses was conducted to establish the bivariate associations between each Dark Triad trait and momentary affect. Next, models with all level-2 predictors were analyzed to assess the potential importance of the Dark Triad traits as predictors of momentary affect. We also evaluated the strength of relationships between affective states and dark traits (pseudo R-square) [116] (p. 798). The Benjamini and Hochberg [119] false discovery rate procedure was used to adjust for multiple testing. This method controls the probability that a true null hypothesis is rejected. A false discovery rate (FDR) of 5% was applied.

The IBM SPSS software (version 25) was used to compute descriptive statistics, internal consistency, correlation and factor analyses.

## Results

### Preliminary analyses

Descriptive statistics for study variables and correlations between variables are given in Table 1. Bonferroni correction for multiple testing was applied to these results (six the DT traits correlated with three affect measures, 6*3 = 18, 0.05/18 = 0.0028), resulting in a significance threshold of 0.0028. Correlations between the Dark Triad traits and the particular emotional states are shown in S1 Table. In the beginning, a series of unconditional random intercept models (without predictors at any level) with momentary positive affect (PA), momentary negative affect (NA) and affect balance as outcome variables was performed (within-person: y_ij_ = *β*_*0j*_ + *r*_*ij*_, between-person: *β*_*0j*_ = *γ*_*00*_ + *u*_*0j*_). The results demonstrated that for momentary PA (intra-class correlation coefficient, ICC = 0.34), momentary NA (ICC = 0.44) and affect balance (ICC = 0.31) a significant part of their variance was at the within-person level (range 56–69%). Thus, the application of multilevel analysis was supported.

**Table 1:** Descriptive statistics and correlations between study variables.

Variables	*M*	*SD*	1	2	3	4	5	6	7	8	9	10	11
1 Momentary positive affect	4.63	1.54	-										
2 Momentary negative affect	1.86	1.18	-.36*	-									
3 Affect balance	2.78	2.38	.86*	-.79*	-								
4 Grandiose narcissism	4.69	2.60	.05	.03	.02	-							
5 Leadership/Authority	1.43	1.24	.07	.00	.04	.68*	-						
6 Grandiose Exhibitionism	1.85	1.60	.11	.00	.07	.75*	.20*	-					
7 Exploitativeness/Entitlement	1.42	1.02	-.11	.08	-.12	.55*	.20*	.09	-				
8 Vulnerable narcissism	2.97	0.52	-.18*	.33*	-.30*	.12	.04	0.01	.25*	-			
9 Machiavellianism	3.87	0.65	-.21*	.30*	-.30*	.21*	.06	.08	.33*	.44*	-		
10 Boldness	1.68	0.47	.18*	-.20*	.23*	.49*	.49*	.31*	.18*	-.23*	.01	-	
11 Meanness	0.88	0.54	-.21*	.02	-.15	.15	.05	.03	.28*	.22*	.40*	.08	-
12 Disinhibition	0.76	0.40	-.07	.28*	-.20*	.27*	.13	.23*	.16	.32*	.26*	.03	.13

The momentary variables were aggregated before the analysis. Pearson’s correlation coefficient was used. *N* = 270 persons, *n* = 3047 measurements.

* *p <* 0.0028 (two-tailed).

#### Dark traits as predictors of momentary affect

A series of multilevel analyses was conducted to assess bivariate relationships between the Dark Triad traits and momentary affect with the number of the episode (a level-1 predictor) as a control variable (within-person: *y*_*ij*_ = *β*_*0j*_ + *β*_*ij*_ (EPISODE) + *r*_*ij*_) and each Dark Triad trait as a level-2 predictor (between-person: *β*_*0j*_ = *γ*_*00*_ + *γ*_*01*_ (Trait) + *u*_*0j*_, *β*_*1j*_ = *γ*_*10*_ + *u*_*1j*_). The number of the episode was chosen as a control variable because past research using the DRM showed that time of a day is an important predictor of positive and negative affect: across the day, positive affect increases and negative affect decreases [120,121]. Table 2 contains a summary of the analyses for momentary PA, momentary NA and affect balance.

**Table 2:** Multilevel estimates predicting momentary affect from the Dark Triad traits.

	Momentary positive affect				Momentary negative affect				Affect balance			
	*β*	95% CI	*t*	*p*	*β*	95% CI	*t*	*p*	*β*	95% CI	*t*	*p*
Machiavellianism	-0.13*	-0.21, -0.05	3.36	<0.001	0.20*	0.12, 0.28	5.0	<0.001	-0.19*	-0.27, -0.11	5.18	<0.001
Grandiose narcissism	0.03	-0.05, 0.11	0.81	0.36	-0.03	-0.03, 0.09	0.87	0.38	0.005	-0.06, 0.06	0.15	0.88
Leadership/Authority	0.04	0.00, 0.08	1.11	0.27	0.01	-0.03, 0.05	0.27	0.79	0.02	-0.02, 0.06	0.62	0.54
Grandiose Exhibitionism	0.07	0.03, 0.011	1.72	0.086	0.01	-0.03, 0.05	0.17	0.87	0.04	0.00, 0.08	1.03	0.31
Exploitativeness/Entitlement	-0.07	-0.011, -0.03	-1.66	0.097	-0.06	-0.1, -0.02	1.35	0.18	-0.07	-0.11, -0.03	-1.86	0.06
Vulnerable narcissism	-0.11*	-0.19, -0.03	2.72	0.007	0.21*	0.15, 0.27	6.36	<0.001	-0.18*	-0.26, -0.1	5.13	<0.001
Disinhibition	-0.04	-0.12, 0.04	1.0	0.32	0.18*	0.1, 0.26	4.94	<0.001	-0.12*	-0.2, -0.04	3.23	0.001
Meanness	-0.13*	-0.21, -0.05	3.58	<0.001	0.02	-0.06, 0.1	0.59	0.55	-0.10*	-0.18, -0.02	2.53	0.01
Boldness	0.10*	0.02, 0.18	2.81	0.005	-0.10*	-0.18, -0.04	3.13	0.002	0.12*	0.18, 0.06	3.38	<0.001

All the variables were standardized. All the coefficients remained significant after controlling for multiple testing. CI = confidence interval.

* *p <* 0.018 (two-tailed).

Contrary to the expectations, there was no association between grandiose narcissism (and its facets) and momentary affect (Hypothesis 2) and meanness was not related to momentary NA (Hypothesis 6), but it emerged as a negative predictor of momentary PA. Disinhibition positively predicted momentary NA, which was in line with Hypothesis 5. However, it had no relationship with momentary PA, which was inconsistent with Hypothesis 5. The remaining dark traits were associated with both momentary PA and momentary NA, which provided support for Hypotheses 1, 3 and 4. The analyses for Machiavellianism and vulnerable narcissism showed similar results: as predicted, both traits were positive predictors of momentary NA and negative predictors of momentary PA. In turn, the associations of boldness with affect were in the opposite direction: positive for momentary PA and negative for momentary NA. Affect balance was predicted by vulnerable narcissism, Machiavellianism, disinhibition and meanness (negatively) and by boldness (positively). All the above relationships remained significant after controlling for multiple testing by applying the Benjamini-Hochberg correction. The models accounted for 3–5% of the variance in momentary PA, 3–12% of the variance in momentary NA and 4–8% of the variance in affect balance.

Next, the multivariate models with all level-2 predictors were tested to assess the incremental predictive value of the particular Dark Triad traits as predictors of momentary affect, controlling for their shared variance. Again, the number of the episode was a level-1 predictor and all the Dark Triad traits were introduced as level-2 predictors (Table 3). When momentary PA was used as an outcome variable, boldness emerged as a positive predictor (but it lost significance after controlling for multiple testing) and meanness was a significant negative predictor. Machiavellianism, disinhibition and vulnerable narcissism were positively associated with momentary NA, while boldness was negatively associated with momentary NA. However, boldness lost significance after controlling for multiple testing. Finally, when affect balance was introduced as a dependent variable, boldness turned out to be a positive predictor while Machiavellianism emerged as a negative predictor. The overall models accounted for 8% of the variance in momentary PA, 21% of the variance in momentary NA and 16% of the variance in affect balance. Subsequently, the above analyses were rerun with scores on sub-scales of the NPI used instead of overall NPI scores (S2 Table). The facets of grandiose narcissism were not significant predictors of momentary affect.

**Table 3:** Multilevel models predicting momentary affect from the Dark Triad traits.

	Momentary positive affect				Momentary negative affect				Affect balance			
	*β*	95% CI	*t*	*p*	*β*	95% CI	*t*	*p*	*β*	95% CI	*t*	*p*
*Within-person predictor*												
Episode	0.15*	0.11, 0.19	7.2	<0.001	-0.08*	-0.12, -0.04	-3.8	<0.001	0.14*	0.10, 0.17	5.9	<0.001
*Between-person predictors*												
Machiavellianism	-0.08	-0.16, -0.00	-1.9	0.06	0.14*	0.07, 0.21	3.3	<0.001	-0.13*	-0.21, -0.05	-3.1	0.002
Grandiose narcissism	0.03	-0.05, 0.11	0.60	0.55	0.01	-0.09, 0.09	0.2	0.84	0.01	-0.05, 0.07	0.3	0.76
Vulnerable narcissism	-0.04	-0.13, 0.06	-0.79	0.43	0.11*	0.3, 0.19	2.7	0.008	-0.08	-0.16, -0.00	-1.9	0.06
Disinhibition	-0.01	-0.09, 0.07	-0.12	0.9	0.14*	0.07, 0.21	3.3	<0.001	-0.06	-0.14, 0.02	-1.7	0.08
Meanness	-0.10*	-0.18, -0.02	-2.61	0.01	-0.07	-0.15, 0.01	-1.8	0.07	-0.03	-0.11, 0.05	-0.80	0.43
Boldness	0.09	0.01, 0,17	2.17	0.031	-0.09	-0.17, -0.01	-2.0	0.048	0.10*	0.02, 0.18	2.56	0.01

All the variables were standardized. Bold indicates significant values after controlling for multiple testing. CI = confidence interval.

* *p <* 0.018 (two-tailed).

#### Being alone or with people and the relationship between grandiose narcissism and momentary affect

Grandiose narcissism and its facets were not related to momentary affect in the current study. To better understand this result, we performed an additional analysis adding a contextual level-1 variable (ALONE) that determined whether a participant was alone in the assessed situation. Results of past research demonstrated a positive relationship between social activity (i.e., presence of other people) and positive affective states [122]. However, other people are especially important to grandiose narcissists; their presence (or absence) might influence their affective states more compared to non-narcissistic individuals. For example, their narcissistic needs may be fully satisfied only when someone can admire them. Past research showed that narcissistic admiration is negatively related to the preference to be alone [123]. In our analysis ALONE was a dichotomous variable coded -1 for the answer “I was alone” and 1 for the answer “I was with someone else.” First, EPISODE and ALONE were introduced as level-1 predictors of momentary PA (*mPA*_*ij*_ = *β*_*0j*_ + *β*_1j_*(*ALONE*_*ij*_) + *β*_2j_*(*EPISODE*_*ij*_) + *r*_*ij*_). ALONE (*β* = 0.15, *p* < 0.001) and EPISODE (*β* = 0.15, *p* < 0.001) emerged as significant predictors of momentary PA: positive affect was higher when participants were with others and it increased during the day. Second, grandiose narcissism was added to the slope equation for ALONE to test for between-level interaction (*β*_*1j*_ = *γ*_*10*_ + *γ*_11_*(*GN*_*j*_) + *u*_*1j*_). The interaction between grandiose narcissism and ALONE was significant (*β* = 0.04, *p* = 0.03). A coefficient was positive, which means that the ALONE effect on momentary PA was larger when grandiose narcissism was higher. Thus, grandiose narcissism acted as a moderator variable for the relationship between momentary PA and ALONE. However, this relationship lost significance after controlling for multiple testing. Third, the facets of grandiose narcissism were added to the slope equation for ALONE to test for between-level interaction (*β*_*1j*_ = *γ*_*10*_ + *γ*_11_*(*GN-LA*_*j*_) + *γ*_11_*(*GN-GE*_*j*_) *γ*_11_*(*GN-EE*_*j*_) + *u*_*1j*_). The interaction between Grandiose Exhibitionism and ALONE was significant (*β* = 0.04, *p* = 0.025), but it lost significance after controlling for multiple testing. A coefficient was positive: the ALONE effect on momentary PA was larger when Grandiose Exhibitionism was higher. The interactions between Leadership/Authority and ALONE (*β* = 0.01, *p* = 0.65) and between Exploitativeness/Entitlement and ALONE (*β* = -0.01, *p* = 0.76) were not significant. Thus, only one of the facets of grandiose narcissism acted as a moderator variable for the relationship between momentary PA and ALONE.

The moderation analysis was not performed for momentary NA because the relationship between ALONE and momentary NA did not reach significance (*β* = –0.03, SE = 0.02, *p* = 0.09).

## Discussion

The aim of the present study was to examine relationships between affective states in everyday life and dark personality traits. The associations between momentary affect and the Dark Triad were investigated using the DRM, a well-validated instrument for the measurement of daily life experience. This method enables assessing affective states within natural situations during a chosen day of one’s life. In the current study, it was assumed that narcissism and psychopathy were multidimensional constructs. The results provide evidence about the relationships of the Dark Triad with momentary affect, supporting the majority of the predictions.

According to the results, dark traits or their dimensions showed specific associations with momentary affect: momentary PA was positively related to boldness and negatively related to vulnerable narcissism, meanness and Machiavellianism; momentary NA was positively related to vulnerable narcissism, disinhibition and Machiavellianism, and also inversely related to boldness. Affect balance showed associations with boldness (positive) and with vulnerable narcissism, Machiavellianism, disinhibition and meanness (negative). These and other results of the present study are discussed below separately for Machiavellianism, narcissism and psychopathy using evolutionary theory and adaptationist approach to emotions.

### Machiavellianism

When formulating the hypothesis on the association of Machiavellianism with momentary affect, we pointed out the inconsistency between the “cool syndrome” (traditionally considered a main feature of high Machs’ emotionality; [50]) and the results of many studies that revealed the positive correlations of Machiavellianism with neuroticism [e.g., 52]. In the current study, we obtained the predicted positive associations between Machiavellianism and momentary NA, and the negative associations of Machiavellianism with momentary PA and affect balance. These results did not support the conviction about “cold” and “smart” Machiavellians who can control successfully their emotions and “get what they want” form other people (see [50]).

In fact, in the description of the Machiavellian personality made in a classic work by Christie and Geis [50], there seems to be a discrepancy between the above features of high Machs and their very pessimistic view of the world where people are susceptible to manipulation, but they are also cunning and constantly lurking for someone’s mistake or a moment of inattention to achieve their goals at his or her expense. Such a worldview may create constant pressure on Machiavellians who (in their opinion) have to continuously defend themselves against other people. Because the Mach IV scale (in which at least 1/3 of items concerns views on people in general, e.g., “Most people are basically good and kind,” inversely scored) is still used as a measure of Machiavellianism, these negative beliefs are crucial to the assessment although the descriptions of the construct sometimes emphasize only manipulation, not views. On the other hand, one should not be surprised that the inhabitants of the Machiavellian, “dog-eat-dog” world tended to feel more negative and less positive emotions in everyday life, which appeared in our study. According to an evolutionary approach to emotions, “negative emotions motivate the organism to avoid misfortune by escaping, attacking, or preventing harm or by repairing damage” [124] (p. 132), so these emotions seem useful for Machiavellians, constantly surrounded by “enemies.” At the same time, such a tendency may be characterized as lower emotional well-being, which is in line with the results of many studies [e.g., 19,20].

Several current studies have provided arguments supporting the assumption about some kind of emotional vulnerability of people higher in Machiavellianism. In a study by Szijjarto & Bereczkei [39], Machiavellianism was connected with difficulties to express and understand one’s own emotions, but also with emotional instability and ability to experience strong emotions. Inability to express feelings can favor a manipulator. It is due to the fact that it is more difficult for others to catch them. However, it may also cause some costs for a Machiavellian. For instance, this inability can be an obstacle to communication in different situations (not only in close relationships). The recent study [125] has demonstrated the unexpected results, contradictory to the idea of “cold” Machiavellians: Machiavellianism positively predicted break-up distress in romantic relationships. Findings of some other studies may be reinterpreted when the assumed Machiavellian “vulnerability” is taken into account. For example, high Machs tended to engage in cheating only when the risk of being caught is small [126], which can be an effect of high levels of negative emotions experienced. The relationship between Machiavellianism and anxiety sensitivity to social concerns (concern of being rejected by others; [127]) may be partly a result of a Machiavellian view of social life and fear of retaliation. Jonason et al. [17] hypothesized that long-term strategizing (e.g., a delay of gratifications) may be an additional source of stress for Machiavellians, which can be associated with poor health outcomes. The negative relationship between Machiavellianism and various psychological and physical health indicators [17,128,129] is also in line with our hypothesis of Machiavellian vulnerability. In general, negative emotions (conceptualized as defensive mechanisms) can protect Machiavellian individuals from danger and increase their individual fitness. At the same time, this may generate considerable costs for persons higher in Machiavellianism in terms of health and emotional well-being.

### Narcissism

Grandiose narcissism is connected with traits that can promote experiencing positive emotions, such as high self-esteem, extraversion and low neuroticism [46]. However, in our study this dimension of narcissism showed no relationships with momentary affect. Also none of the facets of grandiose narcissism was a significant predictor of affect.

In the present study participants were asked to state whether they were alone or with others in a given situation. Starting from the assumption that being with other people, who can give attention, respect, or admiration, may be more rewarding for the participants with higher grandiose narcissism than for those with lower grandiose narcissism (see [123]), we tested the prediction that grandiose narcissism may serve as a moderator of the association between positive affect and the type of social situation (alone vs. with others). The results provided some support for this prediction: Grandiose Exhibitionism, which is good indicator of narcissistic grandiosity [112], was responsible for this moderation.

The specificity of grandiose narcissism is that narcissistic individuals prefer other people’s company because they constantly seek attention and admiration of others in order to maintain their grandiose self-views [130]. Grandiose narcissists can benefit from experiencing positive affective states in the presence of others because it can help them to avoid catching signals of criticism, a lack of acceptance, or other potential sources of ego threats and enhance the effectiveness of self-presentation (see [131]). Positive affect may help narcissists maintain positive illusions about their own attractiveness, which “may compel narcissists to indiscriminately pursue short-term mating strategy beyond their realistic prospects” [132] (p. 213). Positive emotions shared by individuals build friendship, alliances and family bonds [133]. Moreover, persons who express more positive emotions are rated more positively and people generally prefer interacting with those who have a good mood [134]. Thus, it seems that a tendency to feel more positive emotions while with others can be adaptive for individuals higher in narcissism and increase the effectiveness of the narcissistic strategy.

There has been an unresolved discussion in psychology on whether grandiose narcissism should be treated as an adaptive or maladaptive trait. Our results do not support any conclusions regarding this issue. However, the lack of a main effect of grandiose narcissism (and its sub-dimensions) on momentary PA and momentary NA and a moderating effect of grandiose narcissism (and Grandiose Exhibitionism) on the relationship between being alone or with others and momentary PA encourage us to consider other possible contextual moderators, such as types of situation, communication or interpersonal relationships.

Vulnerable narcissism is defined by such features as neuroticism, anxiety and a tendency to feel high negative affect and low positive affect, and these relationships were replicated in many cross-sectional studies [e.g., 46]. The results of our study provided support to the idea that these tendencies are also observed in everyday life. When considered alone, vulnerable narcissism was relatively the strongest predictor of momentary NA. Additionally, unfavorable affect balance was observed. Since affect is regarded as an important component of subjective well-being, this pattern of relationships prompted the conclusion that this type of a narcissist may pay the highest personal costs related to the emotional aspect of well-being out of all dark personalities due to the emotional vulnerability. On the other hand, narcissistic behavioral strategy is based on exploitation of others; however, vulnerable narcissism is associated with experiencing difficulties in establishing and maintaining interpersonal relationships [135]. Thus, some of these negative emotional states can result in inhibiting the unrealistic aspirations and demands in the name of security (e.g., to prevent the loss of a partner), which can be viewed as adaptive.

### Psychopathy

The triarchic model of psychopathy [73], which was adopted in the current study, proposes boldness (“fearless dominance”,) defined as more “positive” phenotypic expression of fearless temperament, as a dimension of psychopathy. According to the findings of the present study, boldness was the only component of psychopathy (and the only dark trait) that turned out to be positively related to momentary PA and affect balance and negatively related to momentary NA. In other words, only boldness exhibited a pattern of relationships with momentary affect that can be considered psychologically beneficial for a “bold” individual, and that can also be interpreted in terms of higher subjective well-being. The possible biologically adaptive value of positive emotions is also important. Positive emotional states communicate that an individual is safe, healthy, full of energy, so he or she is able to take more risks and make good use of to gain valuable resources. This finding is consistent with earlier studies that demonstrated similar relationships between boldness and a trait positive/negative affect, resiliency [92,89], and well-being [90]. Although boldness is also considered to be connected with diminished physiological and emotional responsiveness [91], our study did not confirm this in relation to positive affective states.

According to our results, disinhibition was associated with momentary NA and negatively with affect balance, so it predicted more negative affective states and unfavorable affect balance. However, momentary PA was not related to disinhibition. The relationship between disinhibition and momentary NA was relatively strong and remained significant after controlling for all the Dark Triad traits. Disinhibition embodies this type of psychopathy that is not related to blunted emotional reactivity [91] but is associated with poor emotional control and irresponsible and impulsive behavior [e.g., 136]. This can lead to situations resulting in distress and negative feelings. However, even persistent negative emotional states can be understood as “an adaptive response to unfavorable circumstances” ([137] p. 100). Thus, taking into account evolutionary functions of emotions, these negative emotional states experienced by disinhibited individuals could prevent them from too risky behavior, which can be beneficial for them (i.e., improve their fitness).

Contrary to the predictions, meanness was not associated with momentary NA. The prediction about negative association between meanness and momentary NA was made based on the characteristics of meanness as callous-unemotional aspect of psychopathy and taking into account the results of previous studies on relationships between this dimension and trait negative affectivity. Meanness as a “callous-unemotional” dimension of psychopathy was connected with deficits in experiencing fear and some other negative emotions [e.g., 138]. However, the findings of other studies on triarchic psychopathy showed different patterns of correlations between meanness and some characteristics associated with negative affectivity. For example, in a study by Brislin et al. [139] no relationship was obtained between trait negative affect and meanness in an incarcerated group, and in a community group this relationship was positive. In a recent meta-analysis [89], despite the fact that triarchic meanness was strongly associated with other models of psychopathy and relevant criteria, it was also positively related to neuroticism, Negative Affectivity as measured by the Personality Inventory for the DSM-5, and internalizing symptoms (anxiety and depression). Additionally, the findings regarding internalizing symptoms turned out highly overlapping for meanness and disinhibition [89]. These meta-analytic findings allow believing that the lack of negative associations between meanness and momentary NA in the current study may be partly the effect of the specificity of measurement of the triarchic meanness. It is also possible that the levels of participants’ meanness were not large enough to demonstrate the expected effects in our group or that the indicators of momentary NA used in the current study were not optimal in the case of meanness as correlations between this psychopathy dimension and particular negative emotional states may be different (e.g., negative for fear and positive for anger).

Meanness turned out to be a negative predictor of momentary PA, which was not anticipated, and remained significant when the Dark Triad traits were considered together. Deficits in experiencing positive emotions are rather not assigned to psychopathy, but some studies showed deficient processing of positive emotional stimuli [138]. The negative relationship between meanness and PA may be also associated with the above-mentioned overlap between triarchic meanness and disinhibition. Overall, our results are in line with the idea that meanness can be connected with poverty of emotional experience, however, our evidence is weak.

A different way to interpret the differences regarding emotions is to analyze more basic personality elements that are behind the particular dark traits and their dimensions [140]. The traits which are shared by all the DT constructs constitute the so-called “dark core” [141,142] that includes Honesty-Humility, disagreeableness [8,143–145], callousness [146], and antagonism [141]. These common features, in themselves, cannot be responsible for differences in emotions. Nevertheless, both the behavior components and other traits may be specific for particular dark personalities. For example, disinhibition, vulnerable narcissism and, to a lesser degree, Machiavellianism are associated with higher neuroticism and introversion [45,52,73], which promotes experiencing negative emotions. Conversely, boldness and grandiose narcissism are related to extraversion, agency, social dominance and high self-esteem [68,73], which can promote positive emotions on different ways [147,148]. However, in the current study, it was the case only for boldness.

### Conclusions and limitations

In summary, we investigated the relationships between the Dark Triad and momentary affective states utilizing an ecologically valid method. Our findings contribute to the literature by clarifying how the Dark Triad traits are related to everyday emotional experience. Different patterns of relationships of momentary PA, momentary NA and affect balance with the dark personality constructs were obtained. The two dimensions of narcissism demonstrated different relationships with daily affectivity and the same was true for the three dimensions of psychopathy and Machiavellianism. The Dark Triad traits explained together a noticeable part of momentary NA variance (21%), but their associations with PA were weaker.

On the basis of our results, only boldness was associated with positive affective states, which seems beneficial to an individual. The participants with higher levels of vulnerable narcissism, disinhibition and Machiavellianism were predisposed to more negative and less positive affect and their affect balance may be seen as unfavorable to them in a given situation. These results can be interpreted in the framework of evolutionary psychology. We speculate that the differences in momentary affect obtained in the current study reflect different behavioral strategies used in daily life by individuals. A tendency to feel negative emotions that was observed in Machiavellian and disinhibited persons and vulnerable narcissists may be conducive to achieving their goals by increasing caution and mistrust in dealing with others, which may reduce the risk of being disclosed and protect against risking too much. In turn, the positive emotions of bold individuals can make it easier to take risks when the situation is favorable whereas the positive emotions of grandiose narcissists (experienced in the presence of others) can make it easier to gain attention, acceptance or admiration.

The current study was the first that investigated everyday affective states in relation to narcissism, Machiavellianism and psychopathy simultaneously. The results confirmed the existence of different patterns of relationships between the Dark Triad traits and momentary affect. The significant overlap between the Dark Triad traits, found in numerous research studies, triggers a discussion whether there is a need of considering all these traits. It is especially important in the case of Machiavellianism and psychopathy because of the “dark dyad” hypothesis [20,149,150] that emphasizes the importance of the similarity between these constructs and their separateness from narcissism. Our results do not support this hypothesis and the idea that Machiavellianism and psychopathy measure the same construct (see [151]) because of the lack of similarity between Machiavellianism and the dimensions of triarchic psychopathy with reference to momentary affect. The relationships of Machiavellianism with momentary affect were congruent with the results for vulnerable narcissism rather than those for psychopathy dimensions. In reference to triarchic psychopathy, the current findings provided support for theory and previous research, confirming the distinctiveness of the three dimensions of psychopathy and the specificity of boldness (as a “positive” psychopathic trait) in the domain of affective functioning. Taken as a whole, the current findings seem to support the appropriateness of multidimensional approach to investigating psychopathy and narcissism as elements of the Dark Triad as a way to deal with the excessive overlap of Machiavellianism and unidimensional psychopathy.

The present study has several limitations. Firstly, it relies on data from a convenience sample of university students, which limits the generalization of the results.

Secondly, all data were obtained from self-report, which has some disadvantages. Personality constructs are commonly measured using self-report questionnaires [152]. To minimize common method biases we applied several techniques recommended by Podsakoff, MacKenzie, Lee and Podsakoff [153]. Well-established and valid questionnaires were chosen to reduce statement ambiguity. Each questionnaire was placed separately with a separate instruction. Participants’ anonymity was preserved in the data collection process, which could reduce social desirability bias. However, multi-method assessment could be valuable for future studies and self-report data should be complemented by informant ratings or behavioral observation [154]. Thirdly, to minimize participants’ burden and increase the accuracy of completing the “diary,” only a few emotional words have been used to assess momentary affect. Future studies should address this issue by using a larger and more representative set of emotional words. Moreover, a dimensional perspective on emotional experience, which was adopted in our study, is only one of the possible perspectives. From an evolutionary point of view, emotions can be understood as solutions to specific ecological problems. Therefore, it would be recommended for future studies to examine relationships between the Dark Triad traits and the particular emotional states using the categorical approach to emotions [155,156]. Fourthly, the relatively low reliability coefficients (Cronbach’s alphas) were obtained for the HSNS and NPI, which can reduce statistical power. Nevertheless, in the current study, the relationships between vulnerable narcissism (HSNS) and affect were significant and consistent with the predictions. Generally, the HSNS is regarded as a well-established and valid measure of narcissistic vulnerability. However, it cannot be excluded that lower reliability of the NPI could attenuate the relationships between the NPI and affect. Fifthly, despite the fact that the DRM was developed to reduce memory biases, it cannot be excluded that such biases could occur and influence the result of the current study [157].

To summarize, in this study relationships between the Dark Triad traits and daily emotional experience were investigated. In general, dark traits (except boldness) were not related to momentary positive affect, but most of them were associated with higher levels of momentary negative affect. In particular, persons higher in Machiavellianism, vulnerable narcissism and disinhibition share a tendency to experience more negative affect during a day. This tendency may lower their subjective well-being, but it can also be interpreted as a defense mechanism protecting them from taking (too) risky actions and decisions.

## Supporting information

S1 Table Correlations between the Dark Triad traits and particular emotions. (DOCX)

S2 Table Multilevel estimates predicting momentary affect from Machiavellianism, vulnerable narcissism, triarchic psychopathy and the facets of grandiose narcissism. (DOCX)
