# Supplementary material for: As cold as a fish? Relationships between the Dark Triad personality traits and affective experience during the day: A day reconstruction study
Source: PLoS One. 2020 Feb 25;15(2):e0229625. doi: 10.1371/journal.pone.0229625 (PMC7041966; doi:10.1371/journal.pone.0229625)
Supplement: S1 Table — (DOCX) [file pone.0229625.s001.docx]

**S1 Table****.** **Correlations between the Dark Triad traits and particular emotions.**

|  | | | happy | relaxed | enthusiastic | depressed | afraid | annoyed |
| --- | --- | --- | --- | --- | --- | --- | --- | --- |
| Spearman's rho | Machiavellianism | coefficient | **-.145*** | **-.152*** | **-.252*** | **.246*** | **.220*** | **.333*** |
|  |  | p-value | *.017* | *.012* | *.000* | *.000* | *.000* | *.000* |
|  | Grandiose narcissism | coefficient | **.162*** | .054 | .012 | -.052 | .041 | .087 |
|  |  | p-value | *.008* | *.377* | *.839* | *.399* | *.507* | *.155* |
|  | Leadership /Authority | coefficient | **.136*** | .059 | .004 | -.062 | -.034 | .066 |
|  |  | p-value | *.025* | *.332* | *.952* | *.310* | *.581* | *.277* |
|  | Grandiose Exhibitionism | coefficient | **.161*** | .054 | .118 | -.043 | .028 | .017 |
|  |  | p-value | *.008* | *.378* | *.053* | *.477* | *.646* | *.782* |
|  | Exploitativeness /Entitlement | coefficient | -.022 | -.011 | **-.164*** | -.002 | .094 | **.128*** |
|  |  | p-value | *.717* | *.857* | *.007* | *.968* | *.125* | *.035* |
|  | Vulnerable narcissism | coefficient | **-.128*** | **-.159*** | **-.153*** | **.358*** | **.333*** | **.303*** |
|  |  | p-value | *.036* | *.009* | *.012* | *.000* | *.000* | *.000* |
|  | Boldness | coefficient | **.230*** | **.166*** | .109 | **-.229*** | **-.146*** | **-.145*** |
|  |  | p-value | *.000* | *.006* | *.073* | *.000* | *.016* | *.017* |
|  | Meanness | coefficient | **-.138*** | -.088 | **-.283*** | .003 | .019 | .039 |
|  |  | p-value | *.024* | *.148* | *.000* | *.955* | *.751* | *.526* |
|  | Disinhibition | coefficient | -.007 | -.096 | -.059 | **.190*** | **.302*** | **.266*** |
|  |  | p-value | *.905* | *.114* | *.336* | *.002* | *.000* | *.000* |

The momentary variables were aggregated before the analysis. Spearman’s correlation coefficient was used. *N* = 270 persons, *n* = 3047 measurements. * *p <* 0.05 (two-tailed).
