# Supplementary material for: As cold as a fish? Relationships between the Dark Triad personality traits and affective experience during the day: A day reconstruction study
Source: PLoS One. 2020 Feb 25;15(2):e0229625. doi: 10.1371/journal.pone.0229625 (PMC7041966; doi:10.1371/journal.pone.0229625)
Supplement: S2 Table — (DOCX) [file pone.0229625.s002.docx]

**S2 Table.** **Multilevel estimates predicting momentary affect from Machiavellianism, vulnerable narcissism, triarchic psychopathy and the facets of grandiose narcissism.**

| DV | Momentary positive affect | | | | Momentary negative affect | | | | Affect balance | | | |
| --- | --- | --- | --- | --- | --- | --- | --- | --- | --- | --- | --- | --- |
|  | ***β*** | **95% CI** | ***t*** | ***p*** | ***β*** | **95% CI** | ***t*** | ***p*** | ***β*** | **95% CI** | ***t*** | ***p*** |
| *Within-person predictor* | | | | | | | | | | | | |
| Episode | **0.15*** | 0.13, 0.17 | 6.67 | <0.001 | **-0.09*** | -0.11, -0.7 | -3.8 | <0.001 | **0.14*** | 0.12, 0.16 | 5.99 | <0.001 |
| *Between-person predictors* | | | | | | | | | | | | |
| Machiavellianism | -0.07 | -0.11, -0.03 | -1.71 | 0.09 | **0.16*** | 0.11, 0.21 | 3.43 | <0.001 | **-0.12*** | -0.08, -0.16 | -3.04 | 0.003 |
| Leadership/Authority | 0.003 | -0.04, 0.04 | 0.08 | 0.94 | 0.04 | 0.00, 0.08 | 0.96 | 0.34 | -0.02 | -0.06, 0.02 | -0.42 | 0.67 |
| Grandiose Exhibitionism | 0.05 | 0.01, 0.09 | 1.33 | 0.18 | -0.01 | -0.05, 0.03 | -0.22 | 0.82 | 0.04 | 0.00, 0.08 | 0.98 | 0.33 |
| Exploitativeness/Entitlement | -0.03 | -0.08, -0.01 | -0.76 | 0.44 | -0.01 | -0.05, 0.03 | -0.24 | 0.81 | -0.02 | -0.06, 0.02 | -0.41 | 0.68 |
| Vulnerable narcissism | -0.03 | -0.08, 0.02 | -0.61 | 0.54 | **0.11*** | 0.07, 0.15 | 2.56 | 0.01 | -0.07 | -0.11, -0.07 | -1.71 | 0.09 |
| Disinhibition | -0.02 | -0.06, 0.02 | -0.26 | 0.80 | **0.12*** | 0.08, 0.16 | 3.32 | 0.001 | -0.07 | -0.11, -0.07 | -1.86 | 0.06 |
| Meanness | **-0.10*** | -0.14, -0.06 | -2.4 | 0.017 | -0.07 | -0.011, -0.03 | -1.83 | 0.07 | -0.03 | -0.07, 0.01 | -0.71 | 0.48 |
| Boldness | 0.09 | 0.05, 0.13 | 2.24 | 0.026 | -0.10 | -0.15, -0.05 | -2.20 | 0.029 | **0.11*** | 0.07, 0.15 | 2.77 | 0.006 |

DV – dependent variable; MACH – Machiavellianism; GN – grandiose narcissism; VN – vulnerable narcissism. All variables were standardized. Bold indicates significant values after controlling for multiple testing (Benjamini-Hochberg correction) CI = confidence interval. * *p <* 0.018 (two-tailed).
